# How media competition fuels the spread of misinformation

**DOI:** 10.1126/sciadv.adu7743

**Published:** 2025-06-18

**Authors:** Arash Amini, Yigit Ege Bayiz, Eun-Ju Lee, Zeynep Somer-Topcu, Radu Marculescu, Ufuk Topcu

**Affiliations:** ^1^ Oden Institute for Computational Engineering and Sciences, The University of Texas at Austin, Austin, TX, USA.; ^2^ Department of Electrical and Computer Engineering, The University of Texas at Austin, Austin, TX, USA.; ^3^ Department of Communication, Seoul National University, Seoul, South Korea.; ^4^ Department of Government, The University of Texas at Austin, Austin, TX, USA.

## Abstract

Competition among news sources over public opinion can incentivize them to resort to misinformation. Sharing misinformation may lead to a short-term gain in audience engagement but ultimately damages the credibility of the source, resulting in a loss of audience. To understand the rationale behind news sources sharing misinformation, we model the competition between sources as a zero-sum sequential game, where news sources decide whether to share factual information or misinformation. Each source influences individuals based on their credibility, the veracity of the article, and the individual’s characteristics. We analyze this game through the concept of quantal response equilibrium, which accounts for the bounded rationality of human decision-making. The analysis shows that the resulting equilibria reproduce the credibility-opinion distribution of real-world news sources, with hyperpartisan sources spreading the majority of misinformation. Our findings provide insights for policymakers to mitigate the spread of misinformation and promote a more factual information landscape.

## Teaser

Game-theoretic modeling shows that media competition over public opinion incentivizes news sources to spread more misinformation.

## INTRODUCTION

Controlling information provides a substantial advantage in political and economic spheres, driving media corporations and political elites into fierce competition. To assert dominance, these actors use various strategies, such as targeting specific audiences or promoting inclusivity by presenting diverse viewpoints. However, some resort to deceptive tactics, including the spread of misinformation, rumors, and sensational narratives, to capture public attention (*1*, *2*). The use of misinformation for political gain is a long-standing practice. For example, Octavian, the founder of the Roman Empire, used propaganda to smear the reputation of his rivals by circulating false stories about them (*3*).

Contrary to the widespread belief that social media is the primary source of misinformation, research indicates that online fake news reaches only a limited audience. In contrast, the widespread exposure and internalization of misinformation among the general population underscore the substantial role of public figures and traditional media channels in its dissemination (*4*). Several factors contribute to this issue, including the inherent news value of fake news, which tends to attract attention (*5*). Beyond its harmful societal consequences, misinformation also poses a strategic risk to its propagators (*6*). Over time, as the public becomes increasingly wary of potential deceit, its response to the information shared by low-credibility sources diminishes. Growing skepticism can lead many to perceive the news source as unreliable, resulting in a gradual loss of interest in its reporting.

Over the past decade, researchers have increasingly studied misinformation, focusing on its production (*4*, *7*, *8*), dissemination (*1*, *5*, *9*), detection (*10*, *11*), and countermeasures (*12*–*15*). However, the influence of news source competition on public opinion and the production of misinformation remain poorly understood. We adopt a distinct approach in comparison to the existing literature. Instead of exploring a single instance of fake news and its spread through social networks (*13*, *16*), we choose to study the long-term effects of frequent misinformation dissemination and its impact on the information landscape.

Despite the continued relevance of traditional media, the rise of social networks has transformed the information landscape (*17*), challenging the monopoly of traditional media. Thus, incorporating social media into competition modeling between news sources is crucial. To model this competition, we use opinion dynamics modeling, which is a widely accepted modeling method for the flow of information and the evolution of individual opinions over time (*18*–*20*). We assume that news sources share information with a constant frequency. This assumption stems from empirical research suggesting that media organizations operate in a continuous cycle, driven by content demand in addition to the occurrence of newsworthy events (*21*, *22*). Thus, although newsworthy events do not occur at a regular rate, news outlets prioritize frequency and predictability in their coverage. To simplify the behavior of news outlets, we model news dissemination as a discrete-time decision-making problem, where each source can decide whether to disseminate misinformation or factual information at each news cycle. While this model is a simplification of the real-world behavior of news outlets, it is sufficiently detailed to model the competition between news outlets for public attention at each news cycle and the trade-offs of spreading misinformation.

We hypothesize that fake news and misinformation have an intrinsic news value, which attracts a broader audience (*4*, *23*). We base the proposed model on the assumption that while the dissemination of fake news can provide short-term attention, its prolonged circulation ultimately erodes community trust, leading the public to question the credibility of news sources (*24*). To model opinion dynamics in scenarios where news sources disseminate misinformation, we first formally define the credibility of a news source based on the probability of choosing a random article from the source in question to be truthful. We then use a mixture of source credibility and the article veracity to determine its influence on individuals.

**Fig. 1.**
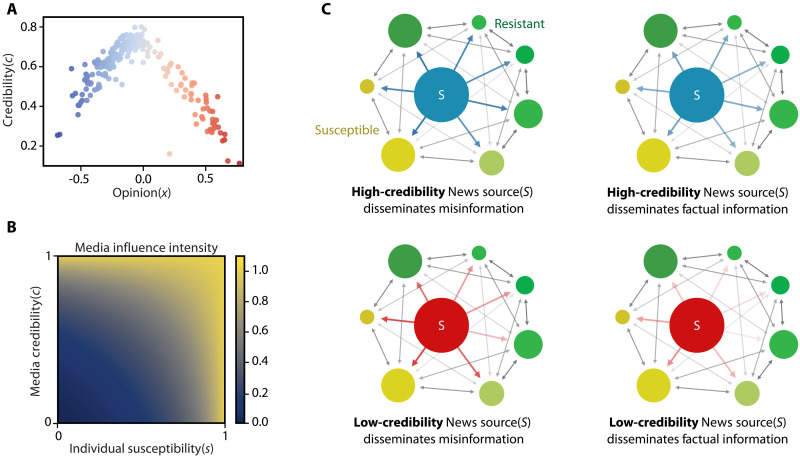
News sources gain additional attention when sharing misinformation. (**A**) Credibility with respect to political bias for 223 news sources from Ad Fontes media bias chart (*59*). The hyperpartisan sources are less credible compared to the centrist ones. (**B**) Normalized media influence intensity with respect to news source credibility and individual susceptibility. Credibility becomes irrelevant for susceptible individuals. (**C**) Visualization of how credibility and disseminating misinformation affect individuals with different levels of susceptibility. Green users are resistant to misinformation, while yellow users are susceptible. The opacity of the arrows increases with increased influence. Sources with high credibility (blue) have notable influence over all users, even when disseminating misinformation. Sources with low credibility (red) have almost no influence over less susceptible individuals; however, disseminating misinformation substantially increases their influence over susceptible individuals.

We assume that articles influence individuals based on their opinion and susceptibility—the ability of individuals to critically assess both the origin and reliability of the news content with which they engage—as well as the source’s credibility, its opinion bias, and the article’s veracity. In our model, individuals are skeptical of both the source’s credibility and the content of the article they receive. As a result, even resistant (low-susceptible) individuals can be influenced by misinformation from credible sources (see Fig. 1, B and C). Although this assumption differs from the classical definition of susceptibility, it accurately reflects the behavior of the general public in consuming news (*25*, *26*).

We analyze how competition between news sources drives misinformation dissemination by modeling interactions as a zero-sum game. In this setup, two players—each controlling a set of ideologically similar sources—strategically determine their information dissemination tactics. We analyze the quantal response equilibrium within this framework, which provides a generalization of the Nash equilibrium that incorporates an additive entropy regularization term to account for randomness and bounded rationality in human decision-making. Our findings indicate that the equilibria highlight four critical phenomena. First, the equilibrium replicates the credibility-opinion distribution of real-world news sources seen in Fig. 1A. In particular, the model predicts that hyperpartisan media disseminates more misinformation compared to centrist ones. Second, it demonstrates existence of an arms race between news sources, i.e., when one player increases sharing misinformation, the equilibrium forces the opposing player to do the same, amplifying the spread of misinformation. Third, it explains how the dissemination of misinformation can contribute to the polarization of the opinion distribution and the formation of echo chambers. Finally, it demonstrates that while reducing public susceptibility and penalizing the media for misinformation can reduce polarization, these measures may not necessarily lower overall community exposure to misinformation.

Our main contributions are threefold:

1) Modeling misinformation dynamics—We introduce a model that examines how the credibility of the news source and the dissemination of misinformation shape public opinion over time. This extends misinformation research beyond isolated fake news incidents to broader, long-term trends.

2) Analyzing competitive incentives—By studying the equilibrium, we reveal how competition among news sources can incentivize misinformation spread, offering insights into how sources determine information integrity.

3) Evaluating policy interventions—We assess potential strategies for policymakers to mitigate opinion distribution polarization and public exposure to misinformation based on equilibrium outcome.

For consistency, it is essential to establish a clear definition for the fake news, misinformation, and news source since these terms have different meanings in various contexts. We define fake news and misinformation as any news or information that is partially or entirely incorrect, sensational, and distributed to manipulate the public. Our definition of a news source includes persons (e.g., political elites on social media) and organizations (e.g., media corporations) that frequently publish content to a mass audience. Therefore, this paper employs news sources rather than media to encompass a wider range of news-sharing platforms.

The paper is structured to first introduce the model in which news sources can disseminate misinformation to gain additional attention at a cost to their credibility. We then examine the competition over public influence by analyzing the underlying equilibrium. Finally, we conclude with a discussion on intervention strategies to foster a more balanced information environment and enhance public discourse.

### Related works

#### Causes and consequences of misinformation

The existing research on the causes of misinformation aims to identify the driving political (*27*), social (*28*), and psychological (*29*) reasons behind misinformation propagation. The oldest treatises discussing the political use of misinformation can be traced back to the Renaissance. However, with widespread news sharing via social media and the abundance of online data, there has been a surge in recent research on the causes of misinformation. Misinformation propagates readily between users over online social networks (*5*, *18*). However, Tsfati *et al.* show that mainstream media also remains to be among the main propagators of misinformation (*4*), necessitating the inclusion of mainstream media outlets’ influence in misinformation models. Recent studies also highlight the role of political elites in community exposure to misinformation on online platforms (*30*, *31*), and how it spreads (*1*). Understanding the mechanisms and motivations behind the spread of misinformation by different entities is still in its infancy (*4*). Additionally, research on recommendation algorithms demonstrates their role in shaping network structures, inadvertently reinforcing echo chambers that amplify misinformation (*32*). To this end, we use game theory and opinion dynamics models to provide a mathematical explanation for the stakeholders’ decision-making and why they resort to sharing misinformation.

Reflexivity, the ability of users to reflect on the source and accuracy of the news they consume, is one of the main factors in misinformation propagation that is more influential than partisanship (*29*). Our model incorporates reflexivity as an innate susceptibility parameter, which describes how much each user gets influenced by low-credibility sources and misinformation. The innate susceptibility of users is the main driving factor in misinformation propagation in nonpolitical topics such as COVID-19 rumors (*33*, *34*). Nevertheless, political leaning is still a factor in the propagation of politics-related misinformation, as users tend to receive news from sources that are close to their opinions (*35*, *36*).

#### Game theory

Game theory provides a mathematical framework for studying interactions in a competitive environment (*37*). Recent advances in sequential decision-making and reinforcement learning enabled the solution of complex Markov games with many players (*38*–*40*). These advancements opened a previously unidentified venue to study decision-making in realistic scenarios such as social interactions. Recent research presents a mathematical model for the motivation behind users sharing misinformation on social media, analyzing through a multiplayer Bayesian game and extensively studying the resulting equilibrium for possible solutions (*23*, *41*, *42*).

Research studies apply evolutionary game theory to optimize strategic interventions by governing bodies to combat misinformation during crises like COVID-19 (*16*). Researchers model misinformation dynamics, demonstrating that interventions such as pre-bunking and accuracy nudges can shift equilibrium outcomes toward reduced misinformation spread (*43*, *44*). Beyond intervention strategies, the concept of misinformation games reveals how misunderstandings of game rules alter strategic outcomes, offering deeper insights into misinformation propagation (*45*). Pre-bunking strategies further enhance resistance by exposing individuals to diluted deceptive tactics, improving media literacy, and resilience against fake news (*46*). Game-theoretic models also examine the interaction between misinformation producers and consumers, showing that engagement often arises from factors beyond accuracy concerns. Misinformation producers strategically mix true and false content to maximize engagement, exploiting cognitive biases and attentional constraints (*42*, *43*).

While existing research examines online misinformation and the cognitive factors driving its spread, the role of competing news outlets in shaping public exposure to misinformation remains underexplored. To address this gap, we propose a mathematical framework that models competitive interactions among news sources and analyzes how misinformation shapes these dynamics.

#### Opinion dynamics

Traditional opinion dynamics models provide insights into social network behaviors, from polarization to consensus. The voter model (*47*) simulates opinion adoption through random interactions, while the DeGroot model describes belief updates based on weighted averages of neighbors’ opinions (*48*). Notable extensions include the Friedkin-Johnson model (*49*), which accounts for individual biases, and the bounded confidence model (*50*), which restricts interactions to those with similar opinions. Although these models focus on microlevel interactions, public opinion is also heavily shaped by news sources such as influencers, political elites, and media corporations (*51*).

Here, we model opinion evolution in a multilayered network, where news sources act as external forces shaping public opinion (*51*). In addition, we introduce a mechanism that captures how disseminating misinformation increases the attention of a news source at the cost of its credibility. By integrating these modifications into established opinion dynamics models and resolving the equilibrium of competition among news outlets, we show that key characteristics of social network evolution such as misinformation exposure distribution and credibility-opinion curve for news sources naturally arise.

Existing studies explore opinion dynamics and learning in social networks, examining how belief systems evolve through Bayesian and non-Bayesian mechanisms and how network structures shape consensus and misinformation spread (*19*, *20*). Recent research investigates the complex interplay between media outlets and social consensus (*52*), showing that different communication strategies can lead to fragmented public opinions (*53*). Some studies also frame competition for public influence as a zero-sum game, analyzing equilibrium outcomes based on available information (*54*).

This paper presents a mathematical model that explains how competition among news sources can incentivize the deliberate spread of misinformation. Rather than analyzing isolated misinformation events on social media, we focus on the sequential decision-making processes of news sources and their long-term impact. By computing game-theoretic equilibria in this competitive setting, we demonstrate that rational decision-making can lead news sources to propagate misinformation. Drawing on these insights, we propose strategic interventions to shift equilibria toward fostering a more reliable and trustworthy information ecosystem.

## METHODS

### Opinion dynamics in presence of misinformation

Various models explain the spread of fake news and rumors on social media (*9*, *55*). Although these models provide an estimate for the spread of a single instance of misinformation—such as how scientific rumors spread in social networks (*9*)—they do not address repetitive exposure to misinformation on individuals. Studies highlight that repetitive exposure to misinformation can create illusory truth for individuals (*56*, *57*), which can lead to the spread of misinformation (*58*). This paper focuses on how news sources strategically use misinformation to capture public attention rather than its reach.

To understand the influence of misinformation on public opinion, we assume that news sources can share misinformation to boost engagement, gaining additional influence over public opinion at the expense of damaging their credibility and diminishing their influence over skeptical individuals over time (*35*). We use three main concepts to model this phenomenon: the source credibility, individual susceptibility, and the persuasive appeal of misinformation. The rapid expansion of social media has transformed communication dynamics and the way individuals consume news, challenging the monopoly traditional media once held over the information ecosystem. In response, we propose a model that accounts for both individual-to-individual and source-to-individual interactions within a population. Figure 1C show the key interactions in the proposed model and demonstrate the differing influence of sources with different credibility spreading factual news or misinformation. Note that the influence of news on individuals differs based on the source credibility and news content, as well as individuals’ susceptibilities. We emphasize that our definition of news source encompasses traditional media, elites, and influential groups frequently disseminating information.

We consider a population of N individuals, each holding opinion $x_{t}^{i}\in X\subset \mathbb{R}$ , interacting among themselves and with M news sources. News sources have fixed opinions $y^{m}$ for all m∈[ M ] . For integer P , we define the set of all positive integers up to P by [P]≔{1,⋯,P} . We denote the opinion of all N individuals and all M sources by vector $x_{t}≔\;\left[x_{t}^{1},\;\cdots ,x_{t}^{N}\right]\in {\mathbb{R}}^{N}$ and $y_{t}≔\;\left[y_{t}^{1},\;\cdots ,y_{t}^{M}\right]\in {\mathbb{R}}^{M}$ , respectively.

#### Source credibility

We assume that the opinions of a news source remain static, and the evolution of individual opinions does not influence it. Sources choose to disseminate factual news or misinformation at each time step. While distributing misinformation can attract a broader audience and potentially gain more influence over individuals due to its sensational nature, it also carries a risk. The public may perceive the news sources that frequently share misinformation as unreliable. This perception of untrustworthiness diminishes their impact, particularly affecting their influence on a skeptical audience.

We define the credibility of a source based on the frequency with which it disseminates misinformation. This definition is consistent with the credibility assessment methodologies used by media evaluator companies, such as Ad Fontes Media and the Media Bias/Fact Check websites (*59*, *60*). These organizations assess the credibility of the news source by randomly sampling the news distributed by each source and evaluating the reliability of the sample information. In the proposed model, source m , in time step t , take action $a_{t}^{m}\in \left\{0,1\right\}$ , where $a_{t}^{m}=0$ indicates disseminating misinformation and $a_{t}^{m}=1$ indicates distributing factual news. We denote the credibility for source m , by $c_{t}^{m}$ , which evolves by the following convex combination rule

$$c_{t+1}^{m}=\lambda c_{t}^{m}+\left(1-\lambda \right)a_{t}^{m}$$ (1)

where λ∈(0 , 1) represents the memory parameter for the community. When λ is close to 1, the public has a strong long-term memory. In this case, the public continues to associate news sources with low credibility for past misinformation, even if their current content is factual. Conversely, when λ takes values close to zero, it suggests that the public tends to forget the actions of news sources quickly. Equation 1 illustrates how disseminating misinformation diminishes credibility, while sharing factual news slightly enhances it. We assume that all news sources are initially deemed credible, with an initial credibility score of $c_{0}^{m}=1$ for every source m∈[ M ].

#### Individual susceptibility

Not everyone evaluates information equally. Some individuals carefully consider a news source’s credibility and the accuracy of an article before forming judgments, while others disregard these factors entirely (*33*). To model individual reflection on sources’ news, we use susceptibility, denoted by $s^{i}\in \left[\;0\;,1\;\right]$ , for each individual i∈[ N ] . Susceptibility quantifies the vulnerability of individuals in the information they receive (*26*). The higher susceptibility, the more likely an individual will be influenced by low-veracity information or less credible sources. For example, an individual with s=1 would accept information only based on its opinion regardless of its source or accuracy, while those with low susceptibility prioritize credibility and accuracy in their assessments. This model captures a realistic spectrum of responses to news and misinformation. Here, we set the individual susceptibility based on beta distribution, i.e., $s^{i}∼B\left(\beta_{1},\beta_{2}\right)$ for all i∈[ N ] , where $\beta_{1},\beta_{2}\geq 1$ are shape parameters. When $\beta_{1}/\beta_{2}=1$ , the resulting probability density function becomes symmetric. $\beta_{1}/\beta_{2}\to \infty$ or $\beta_{1}/\beta_{2}\to 0$ indicates the average shifting toward 1 or 0, respectively.

We assume that individuals consider both a news source’s credibility and the veracity of the article it shares (*61*). Skeptical individuals tend to verify each piece of news independently, but are still more likely to accept misinformation from a credible source than from an unreliable source (*62*). This assumption captures the broad influence of trust in real-world news consumption (*63*). Our model simplifies news consumption by focusing solely on the interplay between source credibility and individual susceptibility. Future work could explore how different individuals process news in more detail, as people generally fall into one of four categories based on their susceptibility to true or false information (*24*): (i) the cynical or obstinate audience, (ii) the gullible audience, (iii) the biased or deluded audience, and (iv) the discerning audience. These groups, in turn, become uninformed, confused, misinformed, or well-informed.

#### Opinion evolution

We model the overall force that influences the opinion of individual i by the result of cumulative interaction forces between other individuals and i (social influence) combined with the forces of news source on i (media influence). We model the evolution of opinion through a discrete stochastic process given by

$$\begin{array}{c} \begin{array}{c} x_{t+1}^{i}=\underset{\text{Social influence}}{\underbrace{\frac{a}{A_{t}^{i}}\sum_{j=1}^{N}\phi \left(|\;x_{t}^{i}-x_{t}^{j}\;|\right)\left(x_{t}^{j}-x_{t}^{i}\right)}}\\ \;+\underset{\text{Media influence}}{\underbrace{\frac{b}{B_{t}^{i}}\sum_{m=1}^{M}\psi \left(|\;x_{t}^{i}-y^{m}\;|,c_{t}^{m},a_{t}^{m},s^{i}\right)\left(y^{m}-x_{t}^{i}\right)}}+\sigma w_{t}^{i} \end{array} \end{array}$$ (2)

where $w_{t}^{i}$ are scalar independent and identically distributed random variables drawn from normal distribution, N(0,1) , and σ>0 denotes the strength of disturbance. The disturbance models unknown interactions within or outside the community that affect an individual’s opinion. Non-negative scalar functions ϕ and ψ model interaction forces for social and media influence, respectively. We normalize the interaction forces to ensure a unitary effect over all individuals by $A_{t}^{i}≔\sum_{j=1}^{N}\phi \left(|\;x_{t}^{i}-x_{t}^{j}\;|\right)$ and $B_{t}^{i}≔\sum_{m=1}^{M}\psi \left(|\;x_{t}^{i}-y^{m}\;|,c_{t}^{m},a_{t}^{m},s^{i}\right)$ . The parameters a,b>0 model the amplitude of media and social influence on individuals, respectively. Various choices of ϕ could recover mainstream models, such as DeGroot model (*48*) for ϕ(r)=1 and bounded confidence (*50*) for $\phi \left(r\right)=1_{\left[\;0\;,\;d\;\right]}\left(r\right)$ . Here, we consider the exponential decay interaction (*64*), i.e., ϕ(r)=exp(−κr) for some κ>0 . We specifically select the exponential decaying interaction function to effectively model homophily—the tendency of individuals with similar opinions to associate with and influence each other more than those with differing views. Therefore, in this model, two individuals with the same opinion exert a notably higher influence on each other’s opinions than those with divergent opinions. This approach reflects the natural propensity for like-minded individuals to reinforce each other’s beliefs, amplifying the homophilic effect on the evolution of opinions.

#### Misinformation impact

Modeling interactions between news sources and individuals is more complex than modeling individual-to-individual interactions, especially given the varying effects of source credibility and article veracity on individuals with different susceptibility levels. Misinformation often attracts a larger audience due to its sensational and controversial nature (*1*, *2*), but it can undermine a source’s credibility, particularly among skeptical individuals. Since credibility strongly influences a source’s impact, especially among skeptics, news sources face a dilemma: expand their influence through misinformation or risk damaging their reputation.

To this end, we model the influence of news sources based on the interplay between the source credibility, article veracity, and individual susceptibility by

$$\psi \left(r,c,a,s\right)=\exp(-\;\hat{\kappa }\;f\left(a\right)g\left(c,s,a\right)r)$$ (3)

where f(a)≔1+ηa for some misinformation gain η>0 and $g\left(c,s,a\right)≔1+\zeta \left(1-\omega_{c}c-\omega_{a}a\right)\left(1-s\right)$ for some credibility gain ζ>0 . The function f models the boost in immediate influence a media has on the public when it disseminates misinformation and sensational news aimed to attract a broad audience. Misinformation gain η determines the additional influence a source gains through sharing misinformation.

We define the function $g\left(c,s,a\right)≔1+\zeta \left(1-\omega_{c}c-\omega_{a}a\right)\left(1-s\right)$ to capture how both the credibility of a news source and the veracity of an article influence individuals. This reflects the tendency of individuals to evaluate both the article itself and the source from which it originates. The weights $\omega_{c}$ and $\omega_{a}$ represent a weighted average based on the news consumption habits of the population constrained by $\omega_{c}+\omega_{a}=1$ . Setting $\omega_{c}=1$ and $\omega_{a}=0$ implies that individuals consider only the credibility of the source. Conversely, $\omega_{c}=0$ and $\omega_{a}=1$ indicate that individuals assess only the content of the article, disregarding the source entirely.

People tend to accept news from sources that have similar opinions (*65*, *66*). We model this effect by letting the media influence multiplier ψ decay exponentially with the distance r between the opinions of the source and the individual. This model ensures that individuals are most influenced by news sources whose opinions align closely with their own. $\hat{\kappa }>0$ indicates the level of influence that news sources have in general over the public. In general, news sources have access to a broader audience, and thus, we can assume $\hat{\kappa }\leq \kappa$.

Figure 1B illustrates the intensity of the media influence with respect to the susceptibility of individuals and the credibility of a news source when $\omega_{c}=1$ and $\omega_{a}=1$ . The susceptibility of individuals becomes irrelevant when the news comes from a highly credible source (  c=1  ). However, information distributed by low-credible sources has less influence on skeptical individuals. Credibility gain ξ reflects the penalty applied to sources with low credibility. This design underscores how the interaction between news source credibility and individual susceptibility shapes the overall effect of media influence.

#### Misinformation exposure

The proposed model considers the possibility of news sources disseminating misinformation. Therefore we can assess individual exposure to misinformation, effectively estimating their reliance on misleading and sensational narratives (*31*). Misinformation exposure measures the probability that an individual is exposed to misinformation shared by sources over time. To this end, we define the misinformation exposure score for each individual, i , during T duration by

$$\Gamma_{i}=\frac{1}{\mathrm{TM}}\sum_{t=0}^{T}\sum_{m=1}^{M}\;e^{-\hat{\kappa }|x_{t}^{i}-y^{m}|}\left(1-a_{t}^{m}\right)$$ (4)

where $e^{-\hat{\kappa }|x_{t}^{i}-y^{m}|}$ measures the chance that individual i hears the story shared by source m.

Through this paper, we assume that the network includes N=500 individuals and M=10 sources unless otherwise stated. The initial distribution is randomly selected from the distribution $\rho_{0}$ , where $\rho_{0}$ is assumed to be uniform with support X . We let η=1 and ξ=2 represent the attention gained through sharing misinformation (misinformation gain) and the attention lost for being perceived as untrustworthy (credibility gain), respectively. The homophily coefficient, for the user-user, is set by κ=20 , and for the user-media by $\hat{\kappa }=5$ , assuming that news sources have a stronger homophilic influence. We let a=b=h , where h=0.1 and noise amplitude is $\sigma =0.1\sqrt{h}$ . We assume that source credibility and article veracity have equal weights on assessments of individuals, i.e., $\omega_{c}=\omega_{a}=0.5$ . The susceptibility is distributed by a beta distribution with shape parameters $\beta_{1}=3$ and $\beta_{2}=2$.

### Public opinion evolution

After a news source shares information, fact-checking based on available information becomes feasible. We assume that information about the credibility of news sources are public and accessible to all stakeholders. We define the state as the information about all individuals’ opinions and sources credibility, represented by $q_{t}=\left[\;x_{t}\;;\;c_{t}\;\right]$ . Realistically, estimating every opinion is infeasible; thus, we assume that news sources can access only the discretized distribution of opinions. This assumption stems from the fact that capturing public opinion typically relies on time-consuming and expensive methods such as surveys and polls, which only estimate opinion distributions. We divide the opinion space into l equal intervals and report the number of users in each interval divided by the population size by $z_{t}\in {\mathbb{R}}^{l}$ . Each source can observe $o_{t}=\left[\;z_{t}\;;\;c_{t}\;\right]$ and decide to disseminate misinformation through strategy $\pi^{m}\left(o_{t}\right)$ , by choosing an action $a_{t}^{m}∼\pi^{m}\left(o_{t}\right)$.

Figure 1A presents the real-world credibility and opinion distributions of 223 news sources collected from the Ad Fontes Media Group’s media bias dataset (*59*). It illustrates a trend where centrally placed sources are deemed most credible, while radical sources are more likely to disseminate misinformation. If news sources adhere to a fixed policy, they will converge to stationary states. In this case, the credibility score represents the probability of sharing factual news, i.e., $c_{\infty }^{m}=\mathbb{P}\left(a^{m}=1\right)$ . Thus, the credibility distribution in Fig. 1A provides a ground-truth stationary policy to which to compare the credibility and opinion distributions of the other possible player policies.

**Fig. 2.**
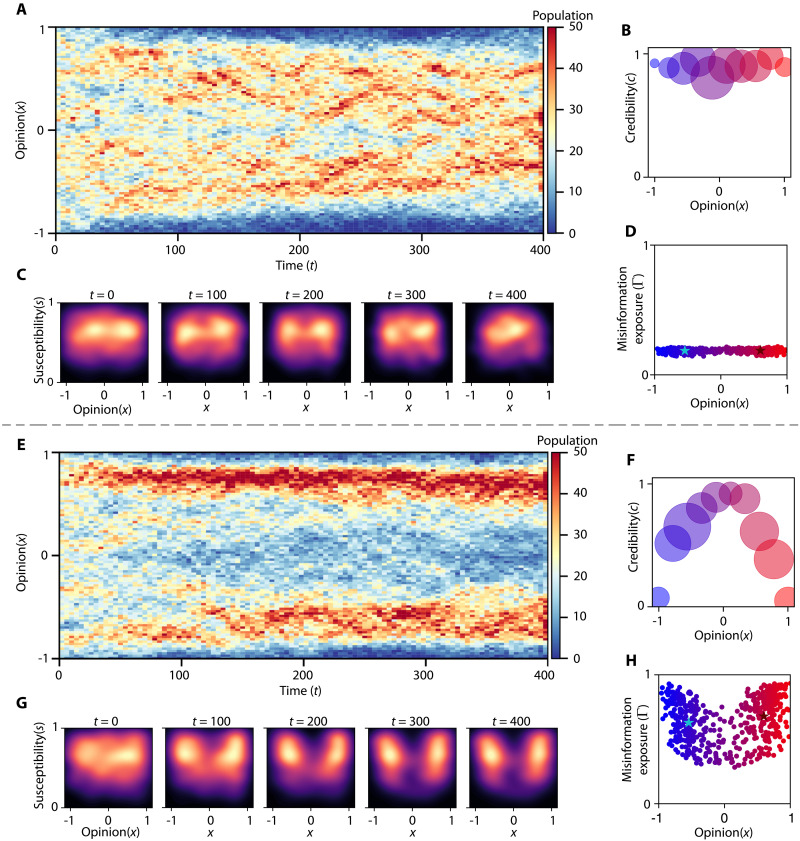
Opinion distribution becomes polarized when news source strategies for disseminating misinformation reflect real-world policies. (**A** and **E**) Evolution of opinions among N=500 individuals corresponding to two different strategies. (**B** and **F**) The radius of the circles correspond linearly to overall media attention. (**C** and **G**) Snapshots of susceptibility and opinion distribution over time steps t={0,100,200,300,400} . (**D** and **H**) Overall misinformation exposure, Γ , with respect to opinion bias. Unbiased individuals are exposed to considerably less misinformation in comparison to others.

Figure 2 shows the distribution of opinions after t=200 steps under a consistent policy $\pi^{m}\left(o_{t}\right)=\pi^{m}$ for all m∈[M] in two scenarios. In the first scenario, news sources strictly limit misinformation, sharing only factual content with occasional errors (Fig. 2, A to D). In the second, hyperpartisan sources spread substantially more misinformation than centrist sources (Fig. 2, E to H). When misinformation dissemination is minimal, public opinion converges toward consensus, reflecting interactions largely free of false information.

Distribution of public opinion becomes polarized when news sources adopt the strategy reported by Ad Fontes Media, as shown in Fig. 1A. Initially, all sources have the same credibility $c_{0}^{m}=1$ for all m∈[M] , making the articles they share seem believable, despite their veracity. At this stage, misinformation affects all users equally, allowing dishonest sources to attract both susceptible and skeptical individuals. As a result, partisan sources quickly establish echo chambers that encompass a broad audience. Over time, as the credibility of these partisan sources declines, skeptical individuals shift toward the center, while susceptible users remain within echo chambers. If skeptical individuals generate enough momentum, this shift can depolarize the community. However, as illustrated in Fig. 2E, even with influence from centrist sources, such migration often fails to prevent stable echo chambers. The results highlight that misinformation can help in forming and sustaining echo chambers, particularly under diverse, nonuniform strategies that mirror real-world dynamics.

Although the proposed model shows that the opinion distribution becomes polarized when news sources share misinformation based on realistic strategies, it does not capture all aspects of polarization. Here, polarization manifests itself primarily as the formation of echo chambers, aligning closely with partisan sorting (*67*). Although the findings highlight misinformation as a possible factor in the polarization of opinion distribution, other factors, such as legal systems, homophily, heterophobia, and individual persistence, may also contribute (*68*).

Figure 2 (B and F) illustrates the final credibility of news sources based on their opinions and their strategies regarding misinformation. The size of each news source reflects the total attention it receives, given by $\sum_{i=1}^{N}\psi_{i,m}$ . In Fig. 2B, where misinformation sharing is limited, the centrist news sources attract the most attention. However, under policies mirroring real world, Fig. 2F shows that partisan news sources dominate media attention, despite their lower credibility. This phenomenon aligns with the observation that partisan news sources can attract more attention than centrist ones, despite having a weaker reputation and a greater tendency to spread misinformation.

In Fig. 2 (D and H), we analyze the final misinformation exposure of the population. When news sources prioritize factual reporting, overall misinformation exposure remains low. Conversely, when source policies reflect real-world scenarios, individuals with radical opinions have substantial exposure to misinformation. These results are consistent with studies on misinformation exposure on platforms like Twitter (*31*), demonstrating that the proposed model effectively captures various aspects of misinformation dissemination. Although the results highlight several phenomena, such as the formation of echo chambers in the presence of misinformation and the distribution of misinformation exposure for individuals, they do not explain why news sources adopt strategies such as those depicted in Fig. 1. Understanding the competitive dynamics among news sources that foster misinformation dissemination is crucial for designing policies to reduce overall misinformation exposure. We delve deeper into the decision-making processes of news sources, exploring how competition for public influence can lead to the spread of misinformation. This investigation will aid in developing more informed and effective media regulations and public policies.

### Equilibrium concept

In the previous section, we discussed the interplay between news sources misinformation dissemination and public opinion evolution. To understand the decision-making processes of news sources in sharing misinformation, we extend this analysis to investigate their strategic behaviors as they compete to increase their influence on public opinion (*23*, *69*). We conceptualize this competition as a zero-sum game, where two players, L, R , each controlling half of the sources, vie for public opinion. Specifically, we consider sources on either side of the political spectrum as coordinated teams seeking to increase their respective parties’ influence.

The player L controls the news sources with $y^{m}<0$ , i.e., $m\in M_{L}=\left\{1,\cdots ,M/2\right\}$ , while the player R controls the sources with $y^{m}<0$ , i.e., $m\in M_{R}=\left\{\;M/2,\cdots ,M\;\right\}$ . We denote $a_{t}^{R}\in A_{R}$ and $a_{t}^{L}\in A_{L}$ as the vector of actions taken by the player R and L , respectively. Each player has $2^{\frac{M}{2}}$ possible actions to choose from and takes actions based on a policy $\pi^{⋆}\left(a^{⋆}|q_{t}\right)$ , where ⋆∈{R,L} . The players want to shift public opinion in their favor by maximizing the population with similar opinions and minimizing those with opposing opinions. We formulate the competition as a zero-sum game, given by

$$\begin{array}{cc} \underset{\pi^{L}}{\text{max}}\;\underset{\pi^{R}}{\text{min}}\; & J≔E\left[\sum_{k=1}^{\infty }\gamma^{k}\;r\left(x_{k},c_{k}\right)\right],\\ s.t.\; & \;\left(2\right),\forall \;i\in \left[\;N\;\right],\\ & \;\left(1\right),\forall \;m\in \left[\;M\;\right],\\ & \;x_{0}∼\rho_{0}\;,\;c_{0}=1 \end{array}$$ (5)

where player L wants to maximize, and player R wants to minimize the cost function J and γ is the discount factor. We draw the initial distribution of opinions, $x_{0}$ , from the uniform distribution $\rho_{0}$ . The players want to steer the public opinion in their direction. Therefore, we define the running cost, $r\left(\cdot ,\cdot \right):{\mathbb{R}}^{N}\times {\mathbb{R}}^{M}\to \mathbb{R}$ , by

$$r\left(x_{t},c_{t}\right)=-\sum_{i=1}^{N}\text{sin}{\left(\varpi x_{t}^{i}\right)}^{\vartheta }$$ (6)

**Fig. 3.**
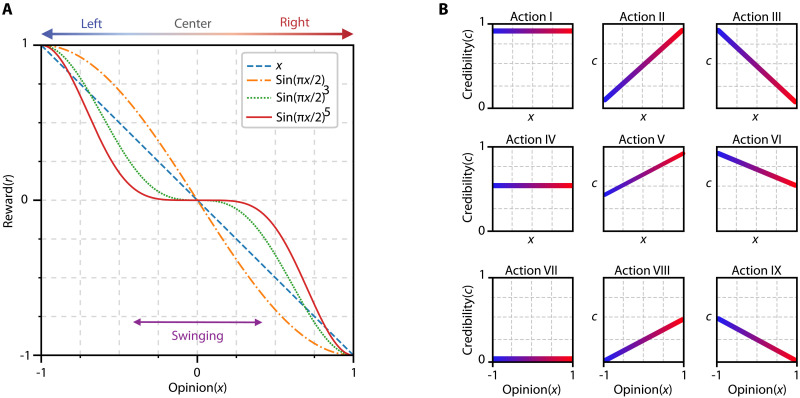
Different candidates for the reward functions and actions. (**A**) The players seek to radicalize public opinion in their favor and minimize the influence of the opponent. The exponent of the reward function controls players’ preference for radicalization. Larger exponents (red, green) indicate a stronger preference for radicalized public opinion. (**B**) Each player selects their actions, from one of the given action templates, to enable scalability.

The parameters $\varpi ≔\frac{\pi }{2}$ and ϑ≔5 represent the strength of the opinion and the steepness target where players aim to change public opinion. Increasing φ shifts the maximum of the reward function toward the center, with the reward peaking at x=1 when $\varpi =\frac{\pi }{2}$ . Meanwhile, ϑ controls the rate of change in the reward function; increasing it leads to a rapid and steep increase or decline in rewards. Figure 3A illustrates various candidates for the reward function. Since the objective of each player is to radicalize the community, individuals who are likely to swing between parties are undesirable as they can shift allegiances effortlessly. To address this aspect, we set ϑ≔5 . This adjustment ensures that the rewards are considerably smaller for individuals in the swinging region and higher for those more radically aligned. This approach incentivizes players to focus their efforts on solidifying and expanding their base of radical supporters, thereby maximizing their influence within these segments. In addition, changing ϖ allows us to tune the level of radicalized players as we aim. Note that the cost function outlined in Eq. (5) does not impose direct penalties on disseminating misinformation or the credibility of news sources. Instead, it indirectly penalizes misinformation dissemination over time through credibility erosion, which manifests as a diminished influence on individuals with low susceptibility.

To measure the polarization of opinion distribution, we use the bimodality coefficient (*70*), ς , defined by

$$\varsigma =\frac{\chi_{3}^{2}+1}{\chi_{4}}$$ (7)

where $\chi_{3}$ and $\chi_{4}$ represent the skewness and kurtosis for the opinion distribution. A bimodality coefficient smaller than $\text{BT}≔\frac{5}{9}$ suggests that the distribution is likely unimodal, indicating little to no polarization of the opinion distribution. Conversely, a coefficient greater than this threshold indicates a multimodal distribution, suggesting substantial polarization. This measure helps to evaluate the effectiveness of sources strategies in influencing public opinion, particularly in terms of polarization.

### Quantal response equilibrium

Human decision-making often deviates from complete rationality (*71*, *72*). Similarly, media corporations and influential groups run by humans can make irrational and sometimes random decisions. Recognizing that the Nash equilibrium does not capture bounded rationality, we adopt the quantal response equilibrium to model the probabilistic nature of human decision-making (*73*, *74*), which provides a generalization of Nash equilibria to scenarios where players necessarily have some randomness in their strategies. For the remainder of the paper, we use the term equilibrium to refer to quantal response equilibrium, unless otherwise stated.

For two given policies μ and μ′ , we define the Kullback-Leibler divergence from μ to μ′ by

$$\text{KL}(\;\mu \;\|\;\mu \prime \;)\;=\sum_{a}\;\mu \left(a\right)\;\text{log}\left(\frac{\;\mu \left(a\right)\;}{\;\mu \prime \left(a\right)\;}\right)$$

To model bounded rationality, we assume that each player’s preferred policy has a bounded Kullback-Leibler divergence from a reference policy $\bar{\pi }$ . We add rationality constraints to the game (*5*) to model the competition with bounded rationality.

A prominent technique to solve the game with a rationality constraint is to use Lagrange multipliers, that is, integrating the constraint into the objective function and transforming the original problem into an unconstrained equivalent (*40*). We then seek to find the value function through

$$\begin{array}{c} \begin{array}{c} \begin{array}{c} V^{*}=\\ \underset{\pi^{L}}{\text{max }}\underset{\pi^{R}}{\text{min }}E\left[\overset{\infty }{\underset{k=1}{\sum }}\gamma^{k}\;\left(r\left(x_{t}\right)-\frac{1}{\tau_{R}}\text{log}\left(\frac{\pi^{R}\left(a_{t}^{R}|o_{t}\right)}{\bar{\pi }\left(a_{t}\right)}\right)+\frac{1}{\tau_{L}}\text{log}\left(\frac{\pi^{L}\left(a_{t}^{L}|o_{t}\right)}{\bar{\pi }\left(a_{t}\right)}\right)\right)\right] \end{array} \end{array} \end{array}$$ (8)

where $\tau_{R}$ , and $\tau_{L}$ denote the rationality of the players. Because of the high dimensionality of this game, solving it with optimal control techniques is impractical. Therefore, we use the function approximation to solve for the value function, achieving a local minimum that approximates the optimal policies for the game.

While our initial model assumes full state observability, tracking individual opinions in practice is both challenging and ethically problematic. Therefore, we assume in subsequent sections that players have only partial observations, reflecting real-world limitations and ethical concerns. Although this adjustment may shift the equilibrium from its fully observable counterpart, it results in a more realistic model.

#### Limiting action space

Assuming that news sources respond instantly to each observation is impractical, as public opinion is difficult to estimate and polling is infrequent. More realistically, each player periodically adjusts its general policy direction. Moreover, computing equilibria for sources that react immediately poses scalability challenges, as the action space grows exponentially with the number of news sources.

To mitigate the curse of dimensionality, we define a finite set of action templates, as shown in Fig. 3B. This approach simplifies the action space by restricting players to a limited set of actions. We evaluate the equilibrium policies of players over these action templates in two scenarios: (i) players respond immediately to each observation, and (ii) players adhere to a fixed policy throughout the competition. In the latter case, the original Markov game (5) reduces to a matrix game, enabling efficient equilibrium computation and comprehensive sensitivity analysis.

Assuming that $B_{L}$ and $B_{R}$ represent the set of action strategies that the players L and R can use, respectively, as shown in Fig. 3B. We define the zero-sum entropy regularized matrix game for completeness by the following

$$\underset{\mu \;\in \;\Delta \left(B_{R}\right)\;\;\;}{\text{max}}\underset{\nu \;\in \;\Delta \left(B_{L}\right)}{\text{min}}\;\mu^{T}\mathrm{A\nu}+\frac{1}{\tau_{R}}H\left(\mu \right)-\frac{1}{\tau_{L}}H\left(\nu \right)$$ (9)

where $A\in {\mathbb{R}}^{p\times p}$ stands for the payoff matrix and $\mu \;\in \;\Delta \left(B_{L}\right)$ and $\nu \;\in \;\Delta \left(B_{R}\right)$ represent the mixed policies for each player, defined respectively as distributions over probability simplex $\Delta \left(B_{R}\right)$ and $\Delta \left(B_{L}\right)$ . We use Shannon entropy, defined as $H\left(\pi \right)≔-\;\sum_{i}\pi_{i}\text{log}\left(\pi_{i}\right)$ , to account for the bounded rationality of players, where π represents an arbitrary distribution over action space. It is well known that the equilibrium for matrix games is unique and satisfies

$$\begin{array}{c} \left\{\begin{array}{cc} \mu^{*}\left(a\right) & =\frac{\text{exp}\left(\tau_{L}{\left[\mathrm{A\nu}*\right]}_{a}\right)}{\sum_{b}\text{exp}\left(\tau_{L}{\left[\mathrm{A\nu}*\right]}_{b}\right)},\;\text{for}\;\text{all}\;a\in B_{L}\\ \nu^{*}\left(a\right) & =\frac{\text{exp}\left(\tau_{R}{\left[\mathrm{A\mu}*\right]}_{a}\right)}{\sum_{b}\text{exp}\left(\tau_{R}{\left[\mathrm{A\mu}*\right]}_{b}\right)},\;\text{for}\;\text{all}\;a\in B_{R} \end{array}\right. \end{array}$$ (10)

The above game solutions follow as a corollary to theorem 4 by Amos (*75*). We use the extragradient method to find the equilibrium efficiently (*76*), by empirically estimating the payoff matrix through 500 simulations.

## RESULTS

In this section, we present the results of the media resorting to misinformation to gain dominance over public opinion. We present the results for hyperresponsive players who exert complete control over each news source’s actions. Subsequently, we introduce strategies to limit the action space, demonstrating that this approach yields a reasonable approximation of the original equilibrium. We then transform the Markov game into a stochastic matrix game by assuming that players consistently select a dominant action, enabling efficient equilibrium computation. This transformation allows for a comprehensive analysis of the sensitivity of various model factors.

### Equilibrium computation

**Fig. 4.**
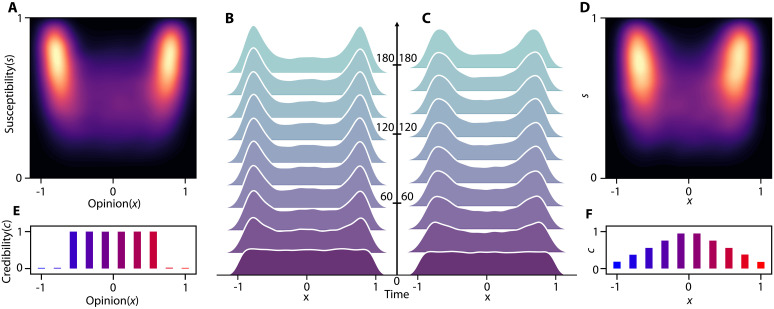
Equilibrium policy incentivizes hyperpartisan media to share more misinformation. The equilibrium for $M^{2}$ actions (**A**, **B**, and **E**) and nine selected action templates (**C**, **D**, and **F**). Both cases entirely polarize public opinion, but the complete action achieves greater separation between peaks. (B) Evolution of opinion when each source can choose any action at each time for the equilibrium. (C) Evolution of opinion distributions when news source actions are limited. [(A) and (D)] Final susceptibility-opinion distribution for complete action and limited strategies, respectively.

Figure 4B illustrates the evolution of the opinion distribution when players, with complete control over the action space, act according to the equilibrium. The equilibrium strategy drives news sources to spread misinformation through hyperpartisan outlets while preserving the credibility of mainstream sources. It exploits the initial high credibility of radical sources to attract individuals early and form echo chambers while maintaining centrist sources’ credibility to influence the opposing party more effectively.

Computing the equilibrium for a large action space presents computational challenges. To address this issue, we use a fixed set of strategies that news sources can choose from. Figure 4C shows the resulting equilibrium when players are limited to actions based on the strategies depicted in Fig. 3B. The equilibrium for both complete and limited action spaces results in a bimodal opinion distribution, and the opinion distribution evolves similarly.

The optimal policy for the case where players can take arbitrary actions, shown in Fig. 4E, drives news source credibility toward either 0 or 1. This contrasts with the real-world credibility distributions in Fig. 1A, where credibility varies smoothly with opinion. We hypothesize two reasons for this discrepancy. First, real-world news sources with similar political ideology often share the same news, allowing misinformation to diffuse among like-minded sources. This diffusion pulls the credibility of similar sources closer, creating a smoother credibility-opinion distribution. Since the proposed model does not account for such inter-source diffusion, it produces sharp transitions in the optimal credibility-opinion distributions.

Second, the complete action space assumes that players have complete control over the actions of all news sources, which is unrealistic. Political parties have only limited influence on media news coverage. Thus, the results for a restricted action set, shown in Fig. 4 (C and D), may better reflect real-world constraints. However, both the complete and limited action cases exhibit a similar trend: Central news sources retain higher credibility, while hyperpartisan sources disseminate most of misinformation. This pattern, also observed in real-world data (Fig. 1A), appears robust to action space limitations. For the remainder of the paper, we assume that players take actions based on the policies in Fig. 3B.

**Fig. 5.**
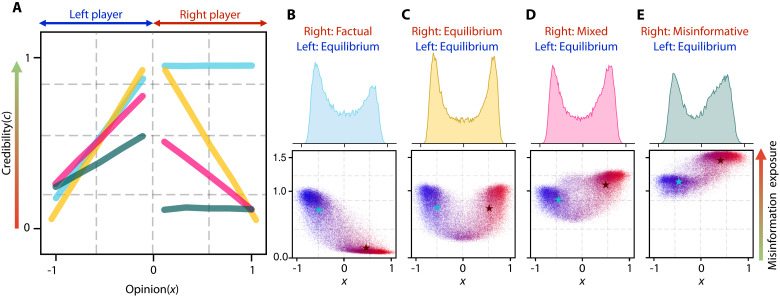
One player disseminating more misinformation incentivizes the other to do the same. (**A**) The right player deviates from equilibrium by spreading more misinformation, while the left player responds optimally. (**B**) The right player avoids misinformation, resulting in a loss of influence. (**C**) Both players act according to equilibrium. (**D**) The right player increases misinformation, forcing the other to lower its credibility. (**E**) The right player exclusively spreads misinformation.

To better understand news sources’ decision-making, we examine scenarios where the right player takes suboptimal actions while the left player follows the optimal policy learned during training. Figure 5 shows that when one player increases misinformation dissemination, the other player’s optimal response is to spread more misinformation as well. Thus, deviating from equilibrium policies in favor of misinformation harms the community in two ways: first, by directly increasing misinformation exposure within its own audience, and second, by prompting the opposing player to lower its credibility, further amplifying the damage. This feedback loop, illustrated in Fig. 5, reveals a reciprocal escalation in misinformation dissemination, resembling an arms race where each player’s actions intensify the other’s strategy.

Our results suggest that the equilibrium in Fig. 5 is stable, meaning that different initial media credibility conditions consistently converge to the same equilibrium state. This stability implies that if one player deviates from the equilibrium, it is encouraged to converge back to taking actions according to the equilibrium policy in Fig. 5C. However, since our analysis relies on function approximation methods, which typically yield local equilibria, more research is needed to validate the uniqueness and robustness of this equilibrium.

### Asymmetrical misinformation exposure

Empirical research on misinformation exposure on Twitter indicates an asymmetric misinformation exposure between parties (*31*). Our findings in Fig. 5 suggest that this asymmetry may stem from differences in strategic use of misinformation. Although this analysis aligns with the observed asymmetry of misinformation exposure on Twitter (X) (*31*), it is not clear why a player might take such suboptimal actions.

#### Asymmetrical initial opinion distribution

**Fig. 6.**
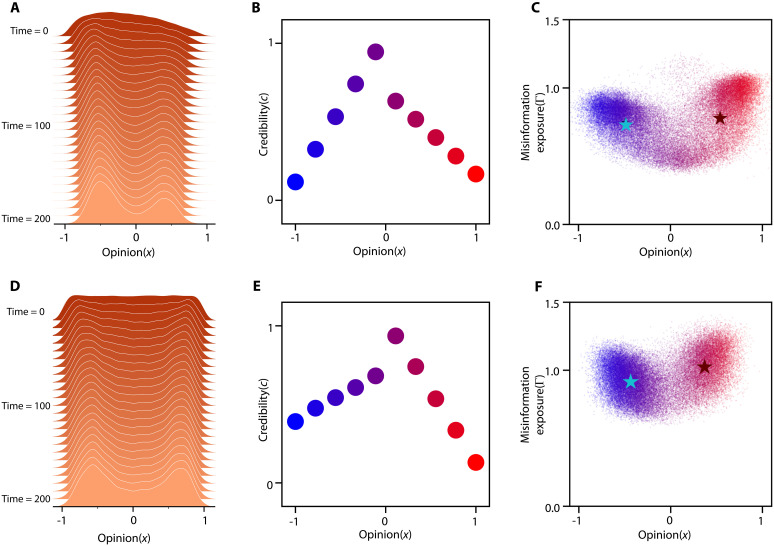
Asymmetric misinformation exposure occurs when one player is at a disadvantage. (**A** to **C**) Equilibrium evolution, under the asymmetrical initial opinion distribution. (A) Both player manage to form a peak. (C) Disadvantageous player share more misinformation compare to the other one. (**D** to **F**) Difference in rationality can lead to asymmetric misinformation exposure. (F) Players with stronger rationality use misinformation more often.

Originally, we assumed that initial opinions were uniformly distributed throughout the spectrum, but this assumption does not always hold. In many cases, one player starts with an advantage, forcing the other to take drastic measures to compensate. Figure 6 (A to C) illustrates such a scenario, where equilibrium is determined under the condition that one side initially has greater popularity. The results show that the less popular player is more likely to resort to misinformation, leading to asymmetry in misinformation exposure.

Despite this disadvantage, the less popular player still establishes a peak in opinion distribution, albeit smaller than the opponent. This observation suggests that strategic use of misinformation can help create a stronghold and prevent assimilation. In particular, the resulting credibility-opinion curve in this scenario more closely resembles real-world observations (Fig. 1A), where one side spreads misinformation more frequently than the other. We emphasize that the proposed model does not capture all aspects of media decision-making, and further empirical studies are needed to draw definitive conclusions.

#### Rationality discrepancy

Another possible explanation for asymmetric misinformation exposure is a difference in player rationality. We define rationality as the degree to which a player avoids taking random actions. An irrational player primarily takes random actions, whereas a more rational player makes decisions to maximize its gains. This difference in rationality can stem from external priorities that influence decision-making in ways unknown to us. In this case, we observe that the more rational player exposes their followers to more misinformation, resulting in a more hyperpartisan community compared to the opponent. Note that the credibility-opinion curve in this scenario (Fig. 6, D to F) differs substantially from the credibility-opinion observed in Fig. 1A. We emphasize that this discrepancy may arise from our assumption that irrational players take random actions. An empirical study is needed to verify whether naive players in such situations act randomly or adhere to specific strategies, such as being entirely honest or dishonest.

### Sensitivity analysis

News sources often follow consistent strategies, particularly before critical events (*77*). Consequently, it is reasonable to assume that each player consistently selects and maintains a predominant strategy throughout the game. This assumption simplifies the Markov game into a matrix game, enabling efficient computation of the equilibrium.

The computational efficiency of matrix games enables us to perform a sensitivity analysis on key factors, including misinformation gain, credibility gain, player rationality, and the distribution of susceptible individuals. This analysis reveals how parameter variations affect game dynamics and outcomes. In addition, it helps predict changes in the information landscape, assisting governments, media, and social elites to combat misinformation effectively.

#### Misinformation and credibility gain

**Fig. 7.**
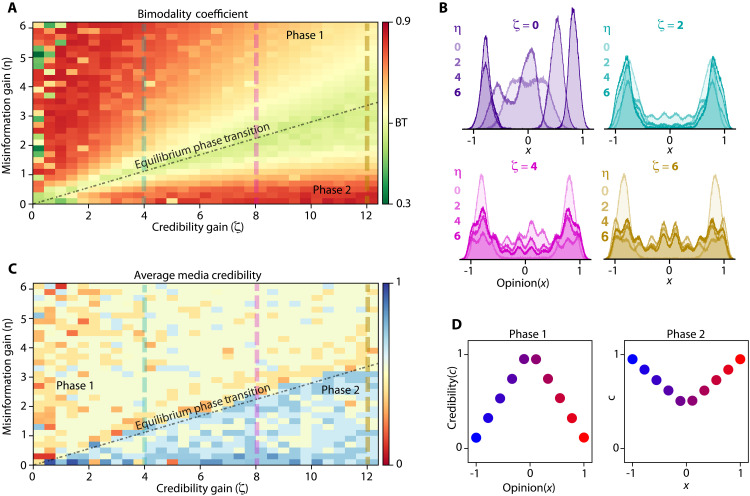
Reducing attention gained from misinformation may cause a phase transition. (**A**) Bimodality coefficient for different values of misinformation gain, η , and credibility gain, ξ . The green region indicates where players cannot exploit policies that lead to polarization. (**B**) Different snapshots of public opinion distributions. (**C**) Average credibility of news sources. Blue indicates more credible. (**D**) Appearance of multiple gains due to misinformation and credibility gain changes.

Recall that ξ , credibility gain, assesses the influence of the credibility of perceived sources on individuals and η , misinformation gain, determines the additional influence gained from disseminating misinformation. Since these parameters enforce the effects of credibility and misinformation on the community, they have a marked impact on the equilibrium. Figure 7 (A and C) shows the bimodality coefficient, ς , and the average credits of news sources, c ≔∑m=1Mcm / M , across various levels of η (misinformation gain) and ξ (credibility gain) at the equilibrium.

When we set both ξ and η to zero, i.e., the actions of the news source are irrelevant, public opinion reaches a consensus. As misinformation gain increases, the optimal policies of players shift toward more aggressive dissemination of misinformation to compete for influence, escalating the polarization of opinion distribution and decreasing the average credibility of news sources. When misinformation gain outweighs credibility gain, the optimal policies mirror the real-world credibility-opinion curve. Increasing the credibility gain or reducing the misinformation gain leads to a phase transition in the equilibrium, as depicted in Fig. 7. During this transition, credibility gain dominates, making it less beneficial for players to rely on misinformation for radicalization; as a result, the equilibrium policies reverse. The players then aim to increase the credibility of their hyperpartisan news source and more frequently disseminate misinformation through centrist sources.

In the second phase, players target low-susceptibility individuals to create echo chambers, contrasting with the first phase, where they rely on susceptible individuals. This observation indicates that in cases where the long-term benefits of remaining credible greatly outweigh the short-term benefits of spreading misinformation, the players can still polarize the opinion distribution of the population by making sure the only available credible news sources are the ones with high biases. Thus, excessive overreliance by the public to the source credibility can also create an information ecosystem that the players can exploit to polarize the opinion distribution. As such, the polarization is lowest around the phase transition region, as can be seen in Fig. 7A.

Our findings suggest that reducing misinformation gain is more beneficial than increasing credibility gain to advocate for a more credible media landscape. While the results advocate for reducing misinformation gain as a more effective strategy, it is essential to acknowledge the practical challenges it presents. Decreasing misinformation gain requires reducing the influence of sources on susceptible individuals, who are the primary audience of partisan news sources. Therefore, for a meaningful change in misinformation gain, one must enforce it from the standpoint of radical sources. However, these changes would weaken the grasp of these sources on susceptible users, which makes such changes difficult to deploy in the real world.

Note that the proposed model does not incorporate all possible aspects that affect opinion distribution polarization. Therefore, changing only misinformation and credibility gain might not be as effective as the results of Fig. 7 predict. However, the emergence of equilibrium policies in such scenarios is dependent on the distribution of public opinion. As a result, despite the effectiveness of the modification of credibility and misinformation gain, the phase transition in Fig. 7 occurs if the community moves toward a unimodal distribution. More research is needed to validate whether the unimodal distribution dictates the phase transition or whether the phase transition itself is a result of the realization of misinformation and credibility gains, and is the cause of the unimodal distribution.

#### Rationality

**Fig. 8.**
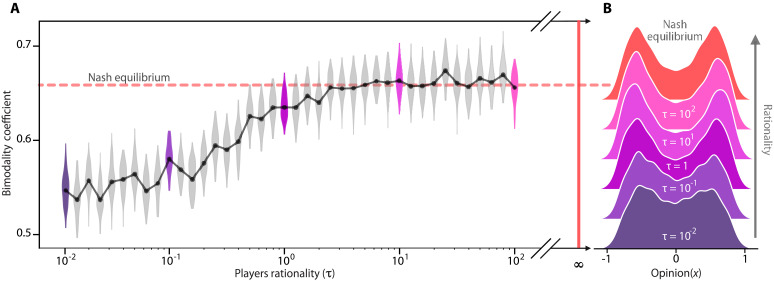
Increasing rationality makes opinion distribution more polarized. (**A**) Bimodality coefficient for different levels of rationality, τ . (**B**) Slices showing opinion distributions of individuals at equilibrium. Increasing τ makes the quantal response equilibrium computation algorithm unstable.

Rationality has a substantial influence on the equilibrium dynamics and the strategies players adapt. The quantum response equilibrium enables us to assess the outcomes across varying levels of rationality. Figure 8 demonstrates that increasing rational decision-making—approaching the Nash equilibrium as a benchmark—drives players toward more extreme strategies, amplifying opinion polarization. Greater rationality enables players to exploit hyperpartisan sources more effectively and disseminate misinformation more frequently, reinforcing their influence.

Crucially, our results indicate that viewing radical sources as less credible is not merely irrational behavior, but a strategic adaptation. Higher rationality improves the effectiveness of misinformation as a tool of influence. The bimodal equilibrium opinion distribution stabilizes for τ>10 , making τ=10 a suitable approximation of the Nash equilibrium while preserving the smoothness required for the convergence of the extragradient method (*76*).

The use of quantal response equilibrium means that we assume fully irrational player to share news with random veracity, i.e., there is an equal chance to share misinformation or factual news. However, further empirical studies are needed to validate this assumption. For instance, one could argue that an irrational player, unconcerned with the reward function, is more likely to share true information, i.e., sharing misinformation only by honest mistakes. Validating such claims is crucial, as they affect the resulting equilibrium and inform effective incentive strategies.

#### Community susceptibility

**Fig. 9.**
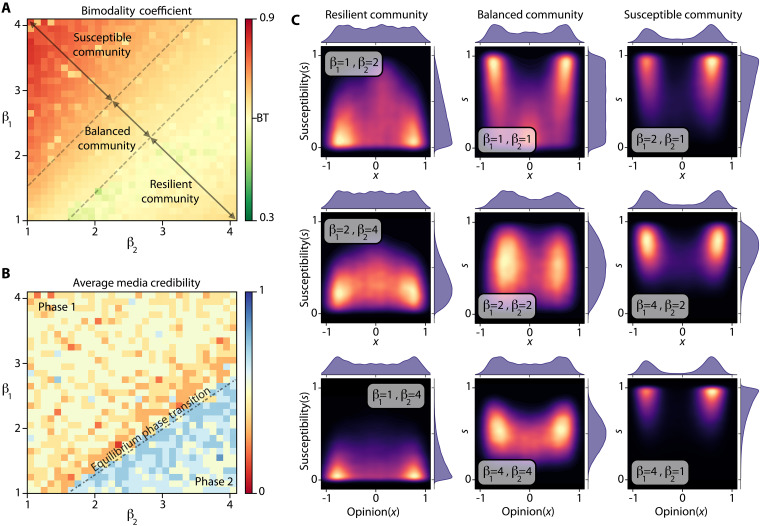
Decreasing community susceptibility can depolarize a susceptible community but results in more misinformation exposure for individuals. (**A** and **B**) Bimodality coefficient and misinformation exposure for different susceptibility distributions. (**C**) Final opinion distributions induced by equilibrium for different susceptibility distributions, with τ=10 , η=1 , and ξ=2 . When a community is resilient to misinformation, players find it difficult to polarize the community.

As the community becomes more resilient to misinformation (i.e., less susceptible), the opinion distribution becomes unimodal. Our results in Fig. 9B show that when moving from a susceptible community to a more balanced community, the average credibility of the media decreases. As a result, in communities with a balanced susceptibility distribution, news sources disseminate more misinformation compared to highly susceptible communities. This phenomenon occurs because in this scenario low-susceptibility individuals form a strong central group and can maintain their population under partisan media sharing more misinformation. Since this central group remains largely unaffected by misinformation, players must attract the remaining predominantly susceptible users by increasing misinformation sharing.

Our results challenge the popular belief that reducing community susceptibility necessarily leads to a more factual information landscape. Instead, it can create a scenario in which the partisan media have greater incentives to use misinformation. However, while the credibility of the news sources decreases in this case, the community shifts toward a unimodal opinion distribution, and consequently, overall misinformation exposure decreases. More empirical studies are needed to explore this trade-off and validate the causal effect of the central skeptical group on the misinformation dissemination strategies of news sources.

Reducing the average susceptibility beyond a certain threshold introduces a phase transition in the equilibrium and results in a sudden shift in average media credibility. Similarly to high credibility gain, in this case, players start to exploit low-susceptible users for opinion distribution polarization, instead of the susceptible users. Even in communities with a balanced susceptibility distribution, the equilibrium often remains in phase 1.

## DISCUSSION

The proposed approach reframes misinformation as a long-term strategic effort to influence public opinion rather than isolated incidents. This perspective recognizes misinformation as an evolving issue shaped by news sources, technologies, and public perception. By modeling it as a dynamic problem, we can leverage control and game theory to predict, regulate, and steer actors toward a more factual information landscape. This ongoing nature of misinformation highlights the need for adaptive, long-term solutions.

Several refinements can enhance the fidelity of our model. For instance, we assume that susceptibility is independent of misinformation exposure, whereas, in reality, repeated exposure can increase susceptibility, further exacerbating misinformation propagation. Another promising direction is to incorporate the sharing of misinformation on social networks to examine its interaction with public opinion. These refinements highlight the value of the model as a tool for informed and responsible decision-making in the information ecosystem.

The sensitivity analysis demonstrates that there are two possible phases for the equilibrium. It indicates that a transition is possible if the attention gained through spreading misinformation or the penalty for low-credible media outlets increases. This transition promote a more factual media landscape and can help to reduce opinion distribution polarization. Therefore, the proposed model allows for investigating the effectiveness of various intervention methods to counter misinformation. This analysis provides public policymakers and government entities with the essential information to make informed decisions about combating misinformation.

### Misinformation interventions

Approaches to counter misinformation (*13*) include debunking (*14*), fact checking (*39*), source accountability (*2*), and enhancing the resilience of the community to misinformation (*26*). Each of these interventions affects specific aspects of the proposed model. Therefore, we can utilize the detailed sensitivity analysis from the previous section to assess the effectiveness of these strategies in countering misinformation and determine when they are most efficient.

#### Debunking misinformation

Fact-checking and debunking, which involve delivering factual information to expose misinformation, are effective tactics for combating misinformation. However, the response of misinformed individuals to such interventions may sometimes be different than expected (*78*). Debunking targets the susceptible part of the community and aims to deliver factual information to fight misinformed individuals. Thus, in our model, the increase in debunking efforts correlates with a reduction in misinformation gain, represented by η . According to the results presented in Fig. 7, the current media landscape is in phase 1, indicating that decreasing misinformation gain is a more efficient tactic than increasing credibility gain.

Debunking requires delivering factual information to community segments heavily exposed to misinformation. The results of Fig. 5C show that radical sources primarily influence these segments. However, most debunking efforts originate from opposing and centrist sources (*79*). Thus, the likelihood of successfully reaching the targeted community is low, making the reduction of misinformation through debunking and fact-checking practically challenging in the current media landscape. Nevertheless, if debunking originates from sources within the same party as the audience, it can effectively reach the misinformed section of the communities, reduce misinformation gain, and shift the equilibrium toward reduced misinformation exposure and polarization.

#### Source accountability

In the proposed model, credibility gain, ξ , enforces how individuals resilient to misinformation are affected by information shared from sources with low credibility. Therefore, holding sources accountable for their actions and stressing their credibility and trustworthiness among the public is equivalent to increasing credibility gain (*80*, *81*). Unlike misinformation gain, which primarily affects susceptible parts of the community, credibility gain affects resilient individuals. Therefore, holding sources accountable can be an effective method to increase credibility gain, especially since the equilibrium in phase 1 positions low-susceptible individuals centrally, where information about the low credibility of radical sources is plentiful. Given that the current equilibrium corresponds to phase 1 in Fig. 7C, this strategy decreases polarization and improves the reliability of news sources. Thus, making media outlets and elites accountable is an efficient solution to counter misinformation, particularly for nonpartisan entities aiming to shift the equilibrium toward a more informed community.

#### Community susceptibility

Our findings underscore the importance of reducing the susceptibility of the community to misinformation. Figure 9B shows that improving susceptibility can lead to the formation of a low-susceptibility group at the center, particularly under a symmetric susceptibility distribution. The presence of this central group incentivizes news sources to spread more misinformation to maintain influence over the remaining and most vulnerable population. The existence of this centrist group not only facilitates mobilization between parties but also dampens the effectiveness of misinformation. As a result, the susceptible portion of the community becomes increasingly hyperpartisan. This dynamic reveals a trade-off between the distribution of community susceptibility and media literacy.

It is also worth noting that our results emphasize the effectiveness of educational initiatives aimed at improving media literacy and lower susceptibility. By equipping individuals with the skills to better assess the credibility of the information they consume, one can decrease the overall susceptibility to the community. While this is an expensive and long-term solution, it discourages the players from sharing misinformation and diminishes its potential to polarize public opinion. Focusing on empowering individuals with knowledge and critical skills is crucial for fostering a more informed and less divided society.

#### Arms race

The experimental results in Fig. 5 expose a critical vulnerability in the current information landscape. When one player lowers its credibility below equilibrium, the optimal response for the opponent is to increase misinformation dissemination. This dynamic underscores how easily adversaries can exploit the system to escalate misinformation.

Consider a scenario in which an adversary introduces a low-credibility source. Regardless of its ideological stance, it will disrupt the equilibrium by initially undermining the perceived credibility of its affiliated party. In response, the opposing party increases misinformation to remain competitive, creating a lose-lose situation for all players.

Conversely, a biased news source with high credibility will improve the equilibrium by prompting competitors to prioritize factual reporting. Promoting or supporting news sources with high credibility, even if they are biased, elevates information quality in the affiliate party and enhances the equilibrium, prompting the other players in the game to compete with high credibility rather than sensationalism.

### Limitations

The proposed model presents an agent-based framework for examining how media competition influences misinformation spread. Our findings demonstrate that competition for public opinion can drive the strategic use of misinformation and incentivize partisan media to share more misinformation. However, the proposed approach has several limitations that warrant further research. Below, we outline these limitations at the news source, theoretical, and societal levels and discuss the scope conditions of our research.

#### News source level

Here, we assume that news sources hold fixed opinion and act to maximize their party’s influence. While this assumption may hold for large media corporations, smaller outlets often shift their opinion in reaction to real-world dynamics. In addition, all news sources pursue financial goals alongside partisan goals, leading to competition even among those aligned with the same belief. Empirical research on the evolution of news sources’ opinion—what political, economic, and social factors shape it and how the public responds to any changes—can be crucial for understanding media decision-making regarding content quality.

Here, we model bounded rationality by assuming that irrational news sources take actions randomly. While this is a typical assumption in game theory, it is unclear whether an irrational news source would randomly decide to share misinformation. One could argue that spreading misinformation requires a level of intent and rationality, meaning an irrational source may only share factual content. Verifying this assumption would require an in-depth empirical study on media decision-making. While media actions are relatively easy to observe, their underlying motivations and strategic interactions remain understudied.

#### Theoretical level

For computational feasibility, we assume that media organizations can only share either factual information or misinformation, recognizing that this simplification does not fully capture real-world media strategies. In practice, misleading content often acts as a precursor to outright falsehoods, strategically blending partial truths with falsity to obscure intent. While misinformation detection has been extensively studied, misleading content is far more difficult to identify, as it deliberately frames facts in a deceptive manner rather than fabricating them entirely.

Our model does not explicitly differentiate between misinformation and misleading content, as misleading narratives may not erode public trust in the same way that clear falsehoods do. Unlike misinformation, which directly damages a media outlet’s credibility, misleading content can preserve perceived credibility while shaping public opinion through selective framing. A more advanced framework that models news-sharing along a continuum—where sources adjust the degree of factual distortion—could provide valuable insights into how media organizations balance attention-seeking strategies with credibility management. Incorporating this distinction would further enhance our understanding of how misleading narratives contribute to misinformation ecosystems without triggering the same reputational consequences as outright falsehoods.

#### Societal level

Here, we narrowed the study scope to an information landscape where two dominant political parties shape media incentives. However, the proposed framework has the potential to extend beyond a two-party system and accommodate more complex information structures. This can be achieved by expanding the opinion dimension and increasing the number of competing players, each optimizing over a more intricate reward function. Exploring these extensions presents an exciting opportunity to examine how different media structures—such as multiparty systems or environments with a central media actor—affect the spread of misinformation. Understanding these dynamics could provide insights into how media architectures influence the strategic dissemination of misinformation and offer pathways to designing information ecosystems that incentivize more truthful and reliable media.

Our results show that misinformation can serve as a tool for partisan gains, and its strategic use may polarize opinion distribution. Notably, polarization has multiple dimensions (*67*, *68*), and further empirical studies are needed to validate misinformation’s role across these dimensions. Our findings demonstrate that the strategic use of misinformation can sort the population based on individual susceptibility, but whether individual susceptibility is the primary factor or not requires further in-depth study.

Last, we emphasize that our model cannot determine whether the strategic use of misinformation results from a deliberate malicious act or the reinforcement of its effects over time. In other words, we cannot explicitly label news sources that strategically use misinformation as malicious, as their decision-making may have emerged through repeated interactions, making information distortion a natural outcome for hyperpartisan media. An in-depth cognitive study could clarify this distinction, determining whether misinformation stems from intentional deception or years of competitive reinforcement. Understanding this phenomenon is crucial due to its considerable impact on designing effective incentives.
